# Novel metaheuristic for parameter estimation in nonlinear dynamic biological systems

**DOI:** 10.1186/1471-2105-7-483

**Published:** 2006-11-02

**Authors:** Maria Rodriguez-Fernandez, Jose A Egea, Julio R Banga

**Affiliations:** 1 Process Engineering Group, Instituto de Investigaciones Marinas (C.S.I.C.), Spanish Council for Scientific Research, C/Eduardo Cabello, 6. 36208 Vigo, Spain

## Abstract

**Background:**

We consider the problem of parameter estimation (model calibration) in nonlinear dynamic models of biological systems. Due to the frequent ill-conditioning and multi-modality of many of these problems, traditional local methods usually fail (unless initialized with very good guesses of the parameter vector). In order to surmount these difficulties, global optimization (GO) methods have been suggested as robust alternatives. Currently, deterministic GO methods can not solve problems of realistic size within this class in reasonable computation times. In contrast, certain types of stochastic GO methods have shown promising results, although the computational cost remains large. Rodriguez-Fernandez and coworkers have presented hybrid stochastic-deterministic GO methods which could reduce computation time by one order of magnitude while guaranteeing robustness. Our goal here was to further reduce the computational effort without loosing robustness.

**Results:**

We have developed a new procedure based on the scatter search methodology for nonlinear optimization of dynamic models of arbitrary (or even unknown) structure (i.e. black-box models). In this contribution, we describe and apply this novel metaheuristic, inspired by recent developments in the field of operations research, to a set of complex identification problems and we make a critical comparison with respect to the previous (above mentioned) successful methods.

**Conclusion:**

Robust and efficient methods for parameter estimation are of key importance in systems biology and related areas. The new metaheuristic presented in this paper aims to ensure the proper solution of these problems by adopting a global optimization approach, while keeping the computational effort under reasonable values. This new metaheuristic was applied to a set of three challenging parameter estimation problems of nonlinear dynamic biological systems, outperforming very significantly all the methods previously used for these benchmark problems.

## Background

Modelling approaches are central in systems biology and provide new ways towards the analysis of omics data, ultimately leading to a greater understanding of the language of cells and organisms [1-3]. Further, these approaches provide systematic strategies for key issues in medicine [4] and the pharmaceutical and biotechnological industries. For example, model-based approaches can provide a rational framework to guide drug development, taking into account the effects of possible new drugs on biochemical pathways and physiology [5]. A common approach to model inter- and intra-cellular dynamic processes is by means of dynamic models, usually consisting of sets of differential equations [6].

The general area of system identification deals with the development of mathematical models of dynamic systems from input/output data [7,8]. When building mathematical models, one starts from the definition of the purpose of the model and uses the a priori available knowledge (i.e. physical, chemical or biological laws, initial hypothesis and/or preliminary data) to choose a model framework and to propose a model structure. This model contain parameters and we need to know whether is it possible to uniquely determine their values (identifiability analysis) and if so, to estimate them with maximum precision and accuracy. This leads to a first working model that must be validated with new experiments, revealing in most cases a number of deficiencies. In this case, a new model structure and/or a new experimental design must be planned, and the process is repeated iteratively until the validation step is considered satisfactory. This iterative process (i.e. the model building cycle) should also contain other elements like optimal experimental design and model discrimination steps [9-13].

This work is focused on the key step of parameter estimation, assuming the structure of the nonlinear dynamic model as given. Parameter estimation (also known as model calibration) aims to find the parameters of the model which give the best fit to a set of experimental data. Examples of recent efforts in the particular case of biochemical pathways are the works of Sugimoto and coworkers [14], Voit and Almeida [15], Rodriguez-Fernandez and coworkers [13] and Polisetty and coworkers [16]. The key issues considered here in this work were to ensure reliable and accurate parameter estimation, paying especial attention to the computational cost, and also to perform the identifiability analysis of the models.

### Parameter estimation in nonlinear dynamic biological models

Estimating the parameters of a nonlinear dynamic model is more difficult than for the linear case, as no general analytic result exists. Biological models are often dynamic and highly nonlinear, thus, in order to find the estimates, we must resort to nonlinear optimization techniques where a measure of the distance between model predictions and experimental data (*Z *= Y˜
 MathType@MTEF@5@5@+=feaafiart1ev1aaatCvAUfKttLearuWrP9MDH5MBPbIqV92AaeXatLxBI9gBaebbnrfifHhDYfgasaacH8akY=wiFfYdH8Gipec8Eeeu0xXdbba9frFj0=OqFfea0dXdd9vqai=hGuQ8kuc9pgc9s8qqaq=dirpe0xb9q8qiLsFr0=vr0=vr0dc8meaabaqaciaacaGaaeqabaqabeGadaaakeaacuWGzbqwgaacaaaa@2DF6@ - *Y*) is used as the optimality criterion to be minimized. The criterion selection will depend on the assumptions about the data disturbance and on the amount of information provided by the user. As explained in detail in the *Methods *section, the maximum likelihood estimator maximizes the probability of the occurrence of the observed measurements. If we make the assumption that the residuals are normally distributed and independent with the same variance *σ*^2^, then the maximum likelihood criterion is equivalent to the least squares and we aim to find p^
 MathType@MTEF@5@5@+=feaafiart1ev1aaatCvAUfKttLearuWrP9MDH5MBPbIqV92AaeXatLxBI9gBaebbnrfifHhDYfgasaacH8akY=wiFfYdH8Gipec8Eeeu0xXdbba9frFj0=OqFfea0dXdd9vqai=hGuQ8kuc9pgc9s8qqaq=dirpe0xb9q8qiLsFr0=vr0=vr0dc8meaabaqaciaacaGaaeqabaqabeGadaaakeaacuWGWbaCgaqcaaaa@2E25@ which minimizes the sum of squared residuals of all the responses. That is, the estimation criterion would be to minimize the *trace *of *Z*^*T *^*Z *[17]. This is subject to the dynamics of the system, plus possibly other algebraic constraints, and model parameters are also subject to upper and lower bounds. This formulation is that of a non-linear programming problem (NLP) with differential-algebraic (DAEs) constraints. In this work, we have followed the so-called single shooting approach [18], where an initial value problem (IVP, i.e., the systems dynamics) is solved as an inner problem of the master NLP problem. When estimating parameters of dynamical systems a number of difficulties may arise, like e.g. convergence to local solutions if standard local methods are used, very flat objective function in the neighborhood of the solution, over-determined models, badly scaled model functions or non-differentiable terms in the systems dynamics [18].

Due to the nonlinear and constrained nature of the systems dynamics, these problems are very often multimodal (non-convex). Thus, traditional gradient based methods, like Levenberg-Marquardt or Gauss-Newton, may fail to identify the global solution and may converge to a local minimum when a better solution exists just a small distance away. Moreover, in the presence of a bad fit, there is no way of knowing if it is due to a wrong model formulation, or if it is simply a consequence of local convergence. Thus, there is a distinct need for using global optimization methods which provide more guarantees of converging to the globally optimal solution, as shown in [19]. The importance of using global optimization methods for parameter estimation in systems biology has been increasingly recognized in recent years [16,20-23]. Global optimization methods can be roughly classified as deterministic, stochastic and hybrid strategies. Deterministic methods can guarantee, under some conditions and for certain problems, the location of the global optimum solution. Nevertheless, no deterministic algorithm can solve global optimization problems of the class considered here with certainty in finite time. Actually, computational effort increases very rapidly (often exponentially) with the problem size. Although very significant advances have been recently made [24-26], these methods have a number of requirements about the dynamics of the system, and currently they do not seem to be applicable to problems with a relatively large number of parameters. Stochastic methods are based on probabilistic algorithms, and they rely on statistical arguments to prove their convergence in a weak way. However, many stochastic methods can locate the vicinity of global solutions in modest computational times [27]. Additionally, stochastic methods do not require transformation of the original problem, which can be treated as a black-box.

In our group, and during the last decade, we have compared a number of different stochastic and deterministic global optimization methods. The overall conclusions from these studies indicate that modern evolution strategies have several key advantages over genetic algorithms and simulated annealing, namely better efficiency/robustness ratio, good scaling properties, an inherent parallel nature and an almost self-tuning mechanism for the search parameters of the method. Our tests and comparisons indicate that DE [28] and SRES [29] are one of the most competitive algorithms, with the additional advantage of being able to handle arbitrary constraints if needed. The main problem presented by these methods is that they require too many evaluations of the objective function [19]. In order to surmount this difficulty, we have recently proposed a hybrid method [13] that speeds up these methodologies while retaining their robustness.

The key concept behind hybrid methods is the well known idea of synergy, that is, a mutually advantageous conjunction of distinct elements. There are several non-trivial questions associated with the actual development of such method, namely choosing which methods to combine, and how to structure such combination. Our work is then focused on selecting more efficient stochastic GO methods and designing better hybrid methods in order to improve the ratio efficiency/robustness. Rodriguez-Fernandez and coworkers [13] combined a global and a local optimization method in a sequential, two-phase hybrid method in order to speedup the stochastic global optimization methods while retaining their robustness. However, computational times were still rather significant, especially if one considers its possible application to larger scale problems.

In order to further increase computational efficiency, in the present work we present a novel metaheuristic approach based on extensions of scatter search combined with various local methods. As it will be shown below, this metaheuristic shows speeds up of between one and two orders of magnitude with respect to previous results obtained with the above mentioned methods. Moreover, this method eliminates the delicate task of deciding where to set the switching point from the global to the local method.

### Global optimization: novel metaheuristic

Scatter search (SS) is a population-based method based on formulations originally proposed in the 1960s for combining decision rules and problem constraints, such as the surrogate constraint method. It was first introduced by Glover [30] as a heuristic for integer programming, although it has been extended for other problem classes more recently [31,32]. Scatter search orients its explorations systematically relative to a set of reference points that typically consist of good solutions obtained by prior problem solving efforts.

The justification for choosing this algorithm as the starting framework for our metaheuristic was based on a recent review comparing a number of global optimization solvers over a set of over 1000 constrained GO problems [33], in which a solver based on scatter search proved to be the best among all the stochastic solvers, and the best among all methods for black-box problems. Furthermore, for problems with a large number of decision variables, this solver also proved to be the most reliable.

Scatter search, when the local search is activated, can be defined as a hybrid method since it combines a global search with an intensification phase (i.e. local search). The algorithm uses different heuristics to efficiently choose suitable initial points for the local search, based on merit and distance filters as well as a memory term. This feature helps overcome the problem of switching from global to local search as explained in [13]. The user does not have to worry about stopping the global search and starting the local solver since the algorithm performs this work automatically.

**Figure 1:**
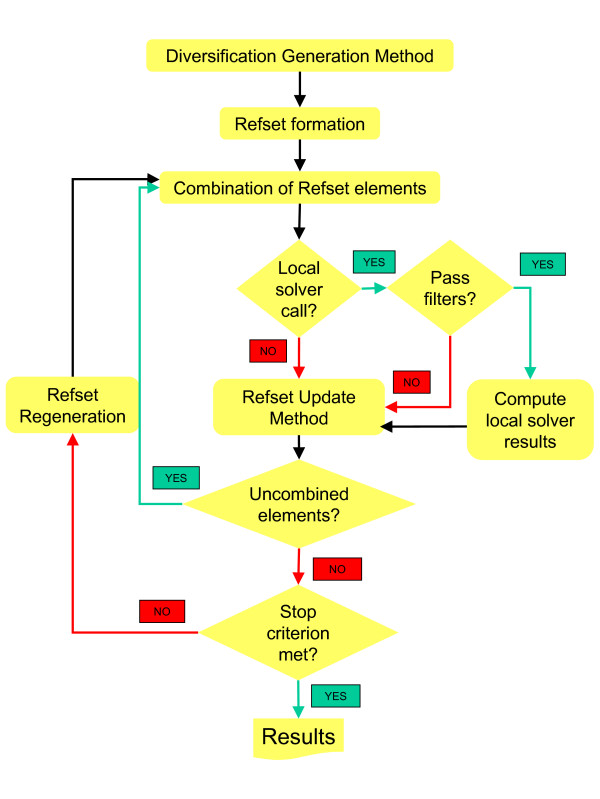
Scatter search pseudo-code diagram.

A scatter search framework in a five-step template is given by Laguna and Martí [31] to describe the basic steps of the algorithm (see Figure 1):

• A *diversification generation method *to generate a collection of diverse trial solutions.

• An *improvement method *to transform a trial solution into one or more enhanced trial solutions.

• A *reference set update method *to build and maintain a reference set consisting of the *b *"best" solutions found (where the value of *b *is typically small, e.g., no more than 20), organized to provide efficient accessing by other parts of the method. Solutions gain membership to the reference set according to their quality or their diversity.

• A *subset generation method *to operate on the reference set, to produce several subsets of its solutions as a basis for creating combined solutions.

• A *solution combination method *to transform a given subset of solutions produced by the *subset generation method *into one or more combined solution vectors.

Of the five methods in the SS methodology, only four are strictly required. The *improvement method *is usually needed if high quality outcomes are desired, but a scatter search procedure can be implemented without it. Differences among scatter search implementations are based in the level of sophistication in which these steps are implemented, not in the presence or absence of other steps. In the algorithm presented here, we have added some advanced features in order to improve its performance when solving parameter estimation problems:

• A logarithmic distribution for generating initial trial solutions can be chosen by the user to favor their presence close to the bounds in terms of Euclidean distance, since the location of the global optimum near or even touching the bounds (i.e. being active at any of the bounds) is quite usual in parameter estimation problems.

• Mechanisms to avoid flat zones (also frequent in parameter estimation problems), as well as others to avoid getting stuck in local solutions, have been added. In every iteration the algorithm analyzes if the elite solutions have very similar objective function values regardless their Euclidean distances. If the variance of these values is too low, our procedure considers that the search is located in a flat zone and resets the elite solutions to explore different (and scattered) areas. This mechanism also prevents the algorithm getting stuck in a local solution when the elite solutions have converged to that minimum.

• A new solution combination method allows to explore deeper the search space. Apart from the traditional method of linear combination between solutions, another method based on building hypercubes around the solutions to generate new solutions inside them has been implemented. Now new points around elite solutions (and not only on the segments joining these elite solutions) can be generated, which favors the intensification and accelerates the convergence.

• When all the combinations among elite solutions have been done, the algorithm can stop or continue by partially rebuilding the set of the elite solutions. A new strategy for rebuilding this set, based in orthogonal search directions has been implemented. Instead of simply maximizing the Euclidean distances between the new elite solutions to be generated and the remaining ones, the algorithm takes into account the directions generated by every pair of elite solutions and force the generator to build new solutions that create new search directions.

• The user can choose a number of different local solvers such us SQP methods like fmincon (The MathWorks Inc.), SOLNP [34], SNOPT [35], direct methods like Nomad [36] for the cases of very noisy data, and others specifically designed for parameter estimation problems such as N2FB/DN2FB [37].

It is interesting to observe similarities and contrasts between scatter search and the original genetic algorithm proposals. Both are instances of what are sometimes called "population based" or "evolutionary" approaches. Both incorporate the idea that a key aspect of producing new elements is to generate some form of combination of existing elements. However, genetic algorithm approaches are predicated on the idea of choosing parents randomly to produce offspring, and further on introducing randomization to determine which components of the parents should be combined. By contrast, the scatter search approach does not emphasize randomization, particularly in the sense of being indifferent to choices among alternatives. Instead, the approach is designed to incorporate strategic responses, both deterministic and probabilistic, that take account of evaluations and history. Scatter search focuses on generating relevant outcomes without losing the ability to produce diverse solutions, due to the way the generation process is implemented.

A detailed description of the method is given in the *Methodology *section.

### Confidence intervals

Determining the parameter values with the maximum likelihood of being correct is only part of the parameter estimation problem. Moreover, it is equally important to find a realistic measure of the precision of those parameters [38,39].

It should be noted that, unlike for the linear case for which a neat theory already exists, there is no exact theory for the evaluation of confidence intervals for systems which are nonlinear in the parameters. An approximate method based on a local linearization of the output function is often used and was also adopted in this study, thus the confidence region is evaluated as a function of the parameter covariance matrix *C*, based on the Fisher information matrix (see details in the *Methods *section).

However, the confidence intervals obtained with the Fisher method are statistically optimistic due to the use of a linear approximation of the non-linear model in the neighborhood of the best parameter estimates [40].

Alternatively, more robust techniques such as the *jackknife *and *bootstrap *methods produce parameter variances that are more realistic. As a drawback, one should mention that these methods are very computing intensive. Another way to obtain the true confidence region of the parameters in non-linear models is by a systematic exploration of the objective functional for an extensive number of parameter combinations. This is a computing intensive task as well, because the number of evaluations increases as a power function of the number of parameters. Therefore, in this study we will make use of the method based on the FIM.

### Precision of parameter estimates

Many difficulties found during parameter estimation are due to a poor identifiability of the model parameters. Parameter identifiability tests should be performed prior to the estimation process to ensure that the parameter estimation problem is well-posed [11]. The identifiability analysis investigates if the unknown parameters of the postulated model can be estimated in a unique way.

Regarding this problem, we can distinguish between structural and practical (or a posteriori) identifiability [41]. Structural identifiability is a theoretical property of the model structure depending only on the observation function and the input function. The parameters of a model are structurally globally identifiable if, under ideal conditions of noise-free observations and error-free model structure, and independently of the particular values of the parameters, they can be uniquely estimated from the designed experiment [8].

The requirements for global structural identifiability are rather strict, since we can find realistic situations where the parameters are not identifiable according to this definition, but nevertheless they would be identifiable for a reasonably restricted set of all possible parameters. This leads to the definition of local structural identifiability, where the requirement for the parameters is to be identifiable in a *ε *neighborhood of a parameter set. Although necessary, structural identifiability is obviously not sufficient to guarantee successful parameter estimation from real data, and this is when the concept of practical identifiability comes into play. In contrast to the theoretical properties of structural identifiability, the practical identifiability is limited by the finite amount of data and the observational noise. Hence, in the presence of large observation errors and/or few data, no reliable estimate is possible and these parameters are called practical non-identifiable.

Assessing a priori global identifiability is very difficult for nonlinear dynamic models, although techniques based on differential algebra have shown very promising results [42]. However, it has been argued that these techniques have somewhat limited applicability [43,44]. These limitations, taken in conjunction with the need for practical methods, provides a key argument for emphasizing the use of practical identifiability despite its limitations derived from its local nature. The question addressed in the a posteriori or practical identifiability analysis is the following: with the available experimental data, can the parameters be uniquely estimated? Or, in other words, if a small deviation of the parameter set occurs, does this have a great impact on the quality of the fit?

The output sensitivity functions (partial derivatives of the measured states with respect to the parameters), are central to the evaluation of practical identifiability. If the sensitivity functions are linearly dependent the model is not identifiable, and sensitivity functions that are nearly linearly dependent, result in parameter estimates that are highly correlated. An easy way to study the practical identifiability of a simple model is to plot the sensitivity functions calculated for a given parameter set. However, this straightforward procedure becomes intractable when the number of measured states and parameters is of realistic size. In the *Methods *section, a numerical procedure to test practical identifiability based on the Fisher information matrix (FIM), as well as an approximate computation of the correlation matrix, are described.

The correlation matrix measures the interrelationship between the parameters and gives an idea of the compensation effects of changes in the parameter values on the model output. If two parameters are highly correlated, a change in the model output caused by a change in a model parameter can be (nearly) compensated by an appropriate change in the other parameter value. This prevents the parameters from being uniquely identifiable even if the model output is very sensitive to changes in the individual parameters.

In order to perform the practical identifiability analysis, prior knowledge of the model parameters is required. In an experimental situation, the parameters values will not be known a priori, and the identifiability analysis will be an important step in an iterative process involving experimental design and parameter estimation [45].

In this work, the new global optimization metaheuristic described above has been coupled with a computational procedure to check identifiability and related measures. This has resulted in an integrated environment to perform robust parameter estimation and identifiability analysis.

## Results and discussion

In order to evaluate the performance and reliability of the novel metaheuristic presented here, which we will denote SSm (scatter search method), we have considered three challenging benchmark problems of increasing order of complexity. All the computations were carried out using a PC/Pentium 4 (1.80 GHz).

### Isomerization of *α*-pinene

**Figure 2:**
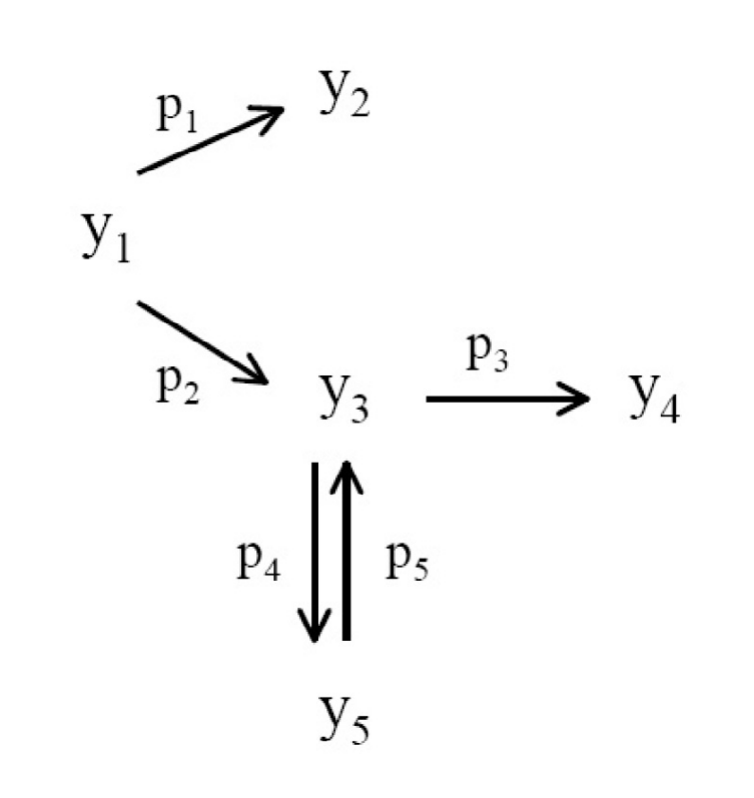
**Mechanism for thermal isomerization of *α*-pinene**. Reaction scheme for the thermal isomerization of a-pinene (*y*_1_) to dipentene (*y*_2_) and allo-ocimen (*y*_3_) which in turn yields *α*- and *β*-pyronene (*y*_4_) and a dimer (*y*_5_).

In this case study, we want to estimate 5 rate constants (*p*_1_,...,*p*_5_) of a complex biochemical reaction originally studied by Box and coworkers [46], which is also part of COPS (Collection of large-scale Constrained Optimization ProblemS) maintained by Dolan and coworkers [47]. Figure 2 contains the proposed reaction scheme for this homogeneous chemical reaction describing the thermal isomerization of *α*-pinene (*y*_1_) to dipentene (*y*_2_) and allo-ocimen (*y*_3_) which in turn yields *α*- and *β*-pyronene (*y*_4_) and a dimer (*y*_5_). This process was studied by Fuguitt and Hawkins [48], who reported the concentrations of the reactant and the four products at eight time intervals (*z*). If the chemical reaction orders are known, then mathematical models can be derived giving the concentration of the various species as a function of time. Hunter and MacGregor [49] assumed first-order kinetics and derived the following linear equations for the five responses:

dy1dt=−(p1+p2)y1     (1)
 MathType@MTEF@5@5@+=feaafiart1ev1aaatCvAUfKttLearuWrP9MDH5MBPbIqV92AaeXatLxBI9gBaebbnrfifHhDYfgasaacH8akY=wiFfYdH8Gipec8Eeeu0xXdbba9frFj0=OqFfea0dXdd9vqai=hGuQ8kuc9pgc9s8qqaq=dirpe0xb9q8qiLsFr0=vr0=vr0dc8meaabaqaciaacaGaaeqabaqabeGadaaakeaadaWcaaqaaiabdsgaKjabdMha5naaBaaaleaacqaIXaqmaeqaaaGcbaGaemizaqMaemiDaqhaaiabg2da9iabgkHiTiabcIcaOiabdchaWnaaBaaaleaacqaIXaqmaeqaaOGaey4kaSIaemiCaa3aaSbaaSqaaiabikdaYaqabaGccqGGPaqkcqWG5bqEdaWgaaWcbaGaeGymaedabeaakiaaxMaacaWLjaWaaeWaaeaacqaIXaqmaiaawIcacaGLPaaaaaa@4375@

dy2dt=p1y1     (2)
 MathType@MTEF@5@5@+=feaafiart1ev1aaatCvAUfKttLearuWrP9MDH5MBPbIqV92AaeXatLxBI9gBaebbnrfifHhDYfgasaacH8akY=wiFfYdH8Gipec8Eeeu0xXdbba9frFj0=OqFfea0dXdd9vqai=hGuQ8kuc9pgc9s8qqaq=dirpe0xb9q8qiLsFr0=vr0=vr0dc8meaabaqaciaacaGaaeqabaqabeGadaaakeaadaWcaaqaaiabdsgaKjabdMha5naaBaaaleaacqaIYaGmaeqaaaGcbaGaemizaqMaemiDaqhaaiabg2da9iabdchaWnaaBaaaleaacqaIXaqmaeqaaOGaemyEaK3aaSbaaSqaaiabigdaXaqabaGccaWLjaGaaCzcamaabmaabaGaeGOmaidacaGLOaGaayzkaaaaaa@3D67@

dy3dt=p2y1−(p3+p4)y3+p5y5     (3)
 MathType@MTEF@5@5@+=feaafiart1ev1aaatCvAUfKttLearuWrP9MDH5MBPbIqV92AaeXatLxBI9gBaebbnrfifHhDYfgasaacH8akY=wiFfYdH8Gipec8Eeeu0xXdbba9frFj0=OqFfea0dXdd9vqai=hGuQ8kuc9pgc9s8qqaq=dirpe0xb9q8qiLsFr0=vr0=vr0dc8meaabaqaciaacaGaaeqabaqabeGadaaakeaadaWcaaqaaiabdsgaKjabdMha5naaBaaaleaacqaIZaWmaeqaaaGcbaGaemizaqMaemiDaqhaaiabg2da9iabdchaWnaaBaaaleaacqaIYaGmaeqaaOGaemyEaK3aaSbaaSqaaiabigdaXaqabaGccqGHsislcqGGOaakcqWGWbaCdaWgaaWcbaGaeG4mamdabeaakiabgUcaRiabdchaWnaaBaaaleaacqaI0aanaeqaaOGaeiykaKIaemyEaK3aaSbaaSqaaiabiodaZaqabaGccqGHRaWkcqWGWbaCdaWgaaWcbaGaeGynaudabeaakiabdMha5naaBaaaleaacqaI1aqnaeqaaOGaaCzcaiaaxMaadaqadaqaaiabiodaZaGaayjkaiaawMcaaaaa@4EDD@

dy4dt=p3y3     (4)
 MathType@MTEF@5@5@+=feaafiart1ev1aaatCvAUfKttLearuWrP9MDH5MBPbIqV92AaeXatLxBI9gBaebbnrfifHhDYfgasaacH8akY=wiFfYdH8Gipec8Eeeu0xXdbba9frFj0=OqFfea0dXdd9vqai=hGuQ8kuc9pgc9s8qqaq=dirpe0xb9q8qiLsFr0=vr0=vr0dc8meaabaqaciaacaGaaeqabaqabeGadaaakeaadaWcaaqaaiabdsgaKjabdMha5naaBaaaleaacqaI0aanaeqaaaGcbaGaemizaqMaemiDaqhaaiabg2da9iabdchaWnaaBaaaleaacqaIZaWmaeqaaOGaemyEaK3aaSbaaSqaaiabiodaZaqabaGccaWLjaGaaCzcamaabmaabaGaeGinaqdacaGLOaGaayzkaaaaaa@3D77@

dy5dt=−p4y3+p5y5     (5)
 MathType@MTEF@5@5@+=feaafiart1ev1aaatCvAUfKttLearuWrP9MDH5MBPbIqV92AaeXatLxBI9gBaebbnrfifHhDYfgasaacH8akY=wiFfYdH8Gipec8Eeeu0xXdbba9frFj0=OqFfea0dXdd9vqai=hGuQ8kuc9pgc9s8qqaq=dirpe0xb9q8qiLsFr0=vr0=vr0dc8meaabaqaciaacaGaaeqabaqabeGadaaakeaadaWcaaqaaiabdsgaKjabdMha5naaBaaaleaacqaI1aqnaeqaaaGcbaGaemizaqMaemiDaqhaaiabg2da9iabgkHiTiabdchaWnaaBaaaleaacqaI0aanaeqaaOGaemyEaK3aaSbaaSqaaiabiodaZaqabaGccqGHRaWkcqWGWbaCdaWgaaWcbaGaeGynaudabeaakiabdMha5naaBaaaleaacqaI1aqnaeqaaOGaaCzcaiaaxMaadaqadaqaaiabiwda1aGaayjkaiaawMcaaaaa@448C@

Assuming this model to be appropriate, the initial conditions for the five species are known, and we can estimate the unknown coefficients *p*_1_,...,*p*_5 _by minimization of a cost function which is usually a weighted distance measure between the experimental values corresponding to the measured variables and the predicted values for those variables. For this problem the cost function can be formulated as:

J(p)=∑j=15∑i=18(yj(p,ti)−y˜ji)2     (7)
 MathType@MTEF@5@5@+=feaafiart1ev1aaatCvAUfKttLearuWrP9MDH5MBPbIqV92AaeXatLxBI9gBaebbnrfifHhDYfgasaacH8akY=wiFfYdH8Gipec8Eeeu0xXdbba9frFj0=OqFfea0dXdd9vqai=hGuQ8kuc9pgc9s8qqaq=dirpe0xb9q8qiLsFr0=vr0=vr0dc8meaabaqaciaacaGaaeqabaqabeGadaaakeaacqWGkbGscqGGOaakcqWGWbaCcqGGPaqkcqGH9aqpdaaeWbqaamaaqahabaGaeiikaGIaemyEaK3aaSbaaSqaaiabdQgaQbqabaGccqGGOaakcqWGWbaCcqGGSaalcqWG0baDdaWgaaWcbaGaemyAaKgabeaakiabcMcaPiabgkHiTiqbdMha5zaaiaWaaSbaaSqaaiabdQgaQjabdMgaPbqabaGccqGGPaqkdaahaaWcbeqaaiabikdaYaaaaeaacqWGPbqAcqGH9aqpcqaIXaqmaeaacqaI4aaoa0GaeyyeIuoaaSqaaiabdQgaQjabg2da9iabigdaXaqaaiabiwda1aqdcqGHris5aOGaaCzcaiaaxMaadaqadaqaaiabiEda3aGaayjkaiaawMcaaaaa@5511@

Box and coworkers [46] tried, in a first instance, to solve this problem without analyzing the multiresponse data, finding parameter values which provided an unsatisfactory data fit. Since ignoring possible dependencies among the responses can lead to difficulties when estimating the parameters (e.g. multiple local minima, very flat objective function, etc.), Box and coworkers described a method for detecting and handling these linear relationships. They showed that there are dependencies in the data and thus only three independent linear combinations of the five responses are used in the identification improving significantly the fit of the data. This analysis of multiresponse data, although efficient, requires a considerable effort specially to uncover the dependencies causes once they have been found, and a deep understanding of the model (that can no longer be considered as a black-box) is essential. Moreover, it becomes unaffordable when the model complexity increases.

Tjoa and Biegler [50] also considered this problem and used a robust local estimation approach to estimate the unknown parameters. They considered the entire data set in order to asses the performance of this method with dependencies in the data, finally reaching the same optimal parameters reported by Box et al. However, the initial value considered for the parameters was very close to the truly optimal solution, which explains why this local method reached the global optimum without getting trapped in a local solution. As pointed out by Averick and coworkers [51], the solution of this problem is not difficult to obtain from initial values of *p *which are close to the global solution, but becomes increasingly difficult to solve from more remote starting points.

In order to avoid the convergence to local solutions without a good initialization value for the parameters and/or further analysis of the multiresponse data, the use of a global optimization approach is proposed here. The lower bounds considered for the five parameters arise from physical considerations, *p*_*i *_≥ 0, and we took the upper bounds to be *p*_*i *_≤ 1, very far from the best known solution (*p*_*1 *_= 5.93e - 5, *p*_2 _= 2.96e - 5, *p*_3 _= 2.05e - 5, *p*_4 _= 27.5e - 5, *p*_5 _= 4.00e - 5). As initial point, we chose *p*_*i *_= 0.5. It should be noted that all the local solvers that we tried with this initial point failed to converge, or converged to bad local solutions.

**Figure 3:**
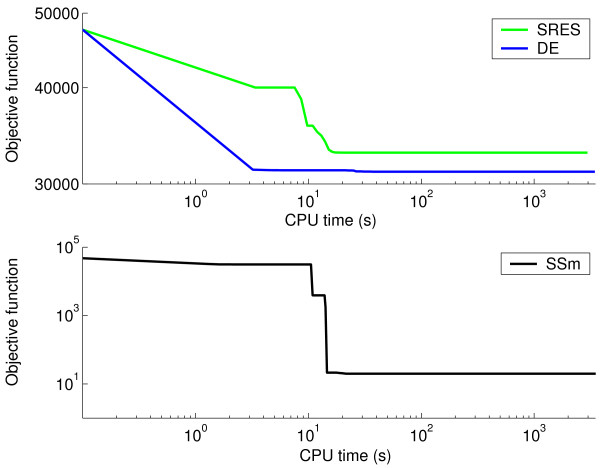
**Convergence curves for the alpha-pinene case study**. Value of cost function versus computation time for SSm, SRES and DE.

Figure 3 (value of cost function versus computation time, the latter in log scale) clearly shows that SSm always converged to the global solution after a short computational time, while two other highly reputed global optimization methods (SRES and DE) failed, or converged in a much larger computational time. In order to help the visualization, the convergence curve corresponding to SSm is represented in a different subplot (with log-log scales), since SRES and DE got trapped in local solutions close to the initial point while SSm converged to the global optimum without difficulties.

**Figure 4:**
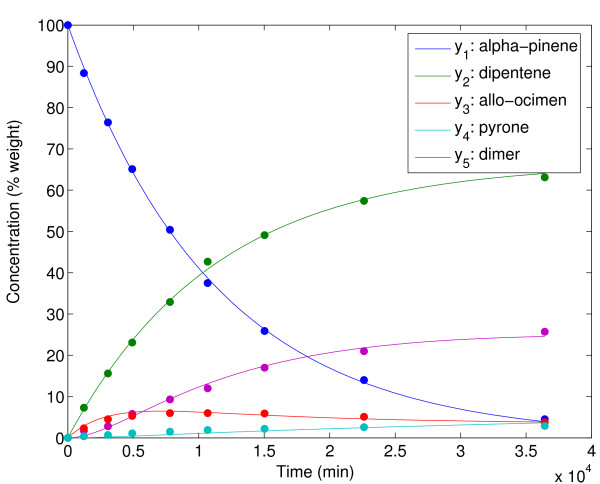
Experimental data versus model prediction for the alpha-pinene case study.

**Figure 5:**
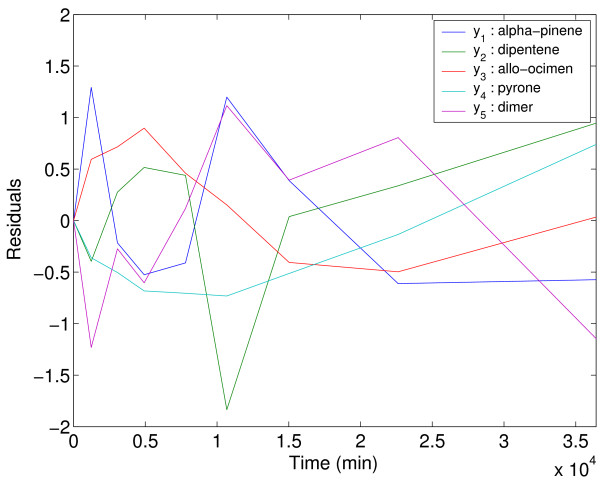
Residuals for the alpha-pinene case study.

Figure 4 shows a comparison between the model predicted values and the experimental data reported by Fuguitt and Hawkins [48] corresponding to the concentration of the reactant and the four products. The estimated parameters allow to reproduce almost exactly the experimental data. Furthermore, the homoscedasticity assumption is confirmed by the lack of correlation between the residuals and time (see Figure 5).

**Figure 6:**
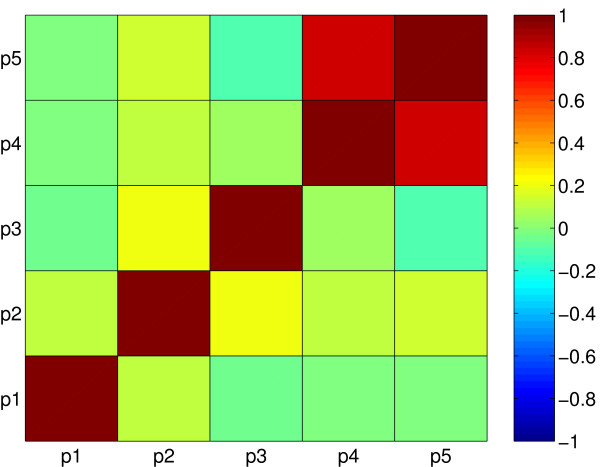
Correlation matrix for the alpha-pinene case study.

The confidence intervals obtained for the optimal parameters, presented in Table 1, are small, indicating a precise estimation. Moreover, the color plot of the correlation matrix in Figure 6 shows a good identifiability at the optimal value with a maximum correlation coefficient of 0.82 between parameters *p*_4 _and *p*_5_. This fact leads us to think that the existence of multiple local minima is the cause of the identification problems experienced in most of the previous studies. These difficulties can be surmounted by proper global optimization methods, as shown here.

**Table 1:** Optimal parameters for the alpha pinene isomerization problem

Optimal parameters (J = 19.87)	
Parameter	Optimal value

*P*_1_	5.9259e-5 ± 1.4391e-6
*P*_2_	2.9634e-5 ± 1.3039e-6
*P*_3_	2.0473e-5 ± 6.6657e-6
*P*_4_	2.7449e-4 ± 5.5314e-5
*P*_5_	3.9980e-5 ± 1.9514e-5

### Inhibition of HIV proteinase

**Figure 7:**
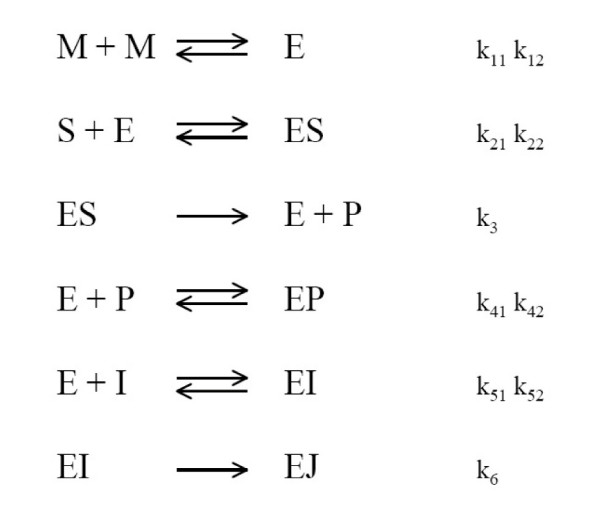
**Mechanism of irreversible inhibition of HIV proteinase**. The HIV proteinase (E) was added to a solution of an irreversible inhibitor (I) and a fluorogenic substrate (S). The enzyme is only active in a dimer form, the product is a competitive inhibitor for the substrate and the inhibitor is irreversible.

This problem consists of the estimation of a number of rate constants of a model for the reaction mechanism of irreversible inhibition of HIV proteinase as originally studied by Kuzmic [52] (Figure 7). The problem considers an experiment where HIV proteinase (assay concentration 0.004 *μ*M) was added to a solution of an irreversible inhibitor and a fluorogenic substrate (25 *μ*M). The fluorescence changes were monitored for 1 h in each of the five experiments conducted at four different concentrations of the inhibitor (0, 0.0015, 0.003, and 0.004 *μ*M in replicate).

We considered the same problem solved by Kuzmic [52] and Mendes and Kell [53] fitting five of the rate constants to the experimental data. In this fit, a certain degree of uncertainty (± 50 %) in the value of the initial concentrations of substrate and enzyme (titration errors) was also assumed. In addition, the offset (baseline) of the fluorimeter was also considered as a degree of freedom. Given that there are five time course curves, there are a total of 20 adjustable parameters: the five rate constants, five initial concentrations of enzyme, five initial concentrations of substrate and five offset values.

By minimization of the sum-of-squares function of the residuals between the measured and the simulated data, the best known solution was obtained by Mendes and Kell using simulated annealing, with a computational cost of 3 million simulations. The next best solution was obtained using a Levenberg-Marquardt method in a considerable shorter computational time (4000 simulations) although this method is only guaranteed to converge to the global minimum if started in its vicinity.

**Figure 8:**
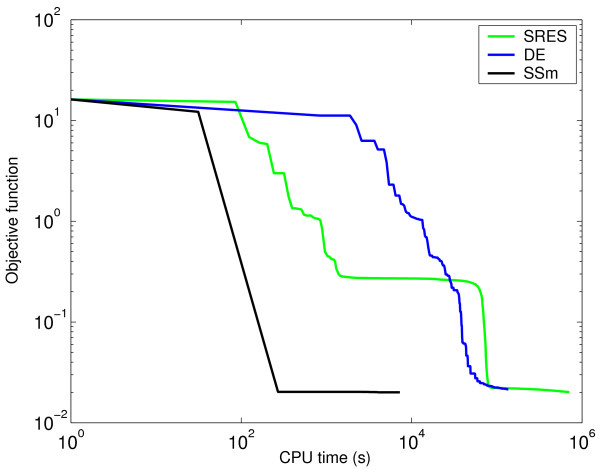
**Convergence curves for the inhibition of the HIV proteinase**. Value of cost function versus computation time (in log scale) for SSm, SRES and DE.

In our study, SSm converged to a better solution in less that 1500 simulations, which confirms the good performance of this method even with challenging parameter estimation problems. Moreover, when compared with other performant stochastic methods such as SRES or DE, SSm reached better solutions with speed-ups of almost 3 orders of magnitude (see Figure 8).

**Figure 9:**
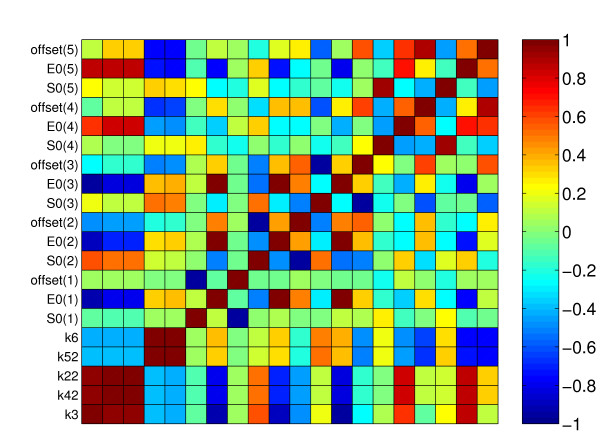
Correlation matrix for the inhibition of the HIV proteinase.

Despite SSm converged in every run to solutions with a very good values of the cost function (always lower than the best value previously published), the values of the parameters were not always the same (see examples in Table 2) indicating a very flat objective function in the region of parameter space near the optimum. The correlation matrix (see Figure 9) helps to explain this fact since there are correlation values of 0.9999 between some pairs of parameters, (like *k*_42 _and *k*_22_) indicating the lack of identifiability for this problem. This characteristic is first reported here and explains the difficulties (i.e. multiple solutions almost equivalent) experienced by previous researches, confirming the importance of coupling identifiability tests with parameter estimation procedures.

**Table 2:** Optimal parameters for the HIV proteinase inhibition problem

Results SSm		
Parameter	Parameter value (J = 0.0199)	Parameter value (J = 0.0203)

*k*_3_	6.235 ± 3.2546	5.656 ± 1.953
*k*_42_	8772 ± 46120	688.4 ± 3436
*k*_22_	473.0 ± 624.6	120.6 ± 508.1
*k*_52_	0.09726 ± 0.1288	4.615 ± 583.4
*k*_6_	0.01417 ± 0.01032	3.531 ± 455.4
*S*_0 _(exp 1)	24.63 ± 0.07817	24.69 ± 0.08049
S_0 _(exp 2)	23.32 ± 1.349	23.43 ± 0.1541
S_0 _(exp 3)	26.93 ± 1.222	27.11 ± 0.1672
*S*_0 _(exp 4)	13.34 ± 1.822	17.07 ± 1.986
*S*_0 _(exp 5)	12.50 ± 1.812	14.49 ± 1.757
*E*_0 _(exp 1)	0.005516 ± 0.001968	0.005397 ± 0.0009091
*E*_0 _(exp 2)	0.005321 ± 0.001309	0.005199 ± 0.0005520
*E*_0 _(exp 3)	0.006000 ± 0.001111	0.006000 ± 0.0005489
*E*_0 _(exp 4)	0.004391 ± 0.00008686	0.004264 ± 0.00005821
*E*_0 _(exp 5)	0.003981 ± 0.00008844	0.003973 ± 0.00005648
offset (exp 1)	-0.004339 ± 0.001788	-0.005611 ± 0.001836
offset (exp 2)	-0.001577 ± 0.002966	-0.004247 ± 0.003432
offset (exp 3)	-0.01117 ± 0.002734	-0.01522 ± 0.003865
offset (exp 4)	-0.001661 ± 0.001881	-0.009649 ± 0.003277
offset (exp 5)	0.007133 ± 0.001764	0.001329 ± 0.003178

**Figure 10:**
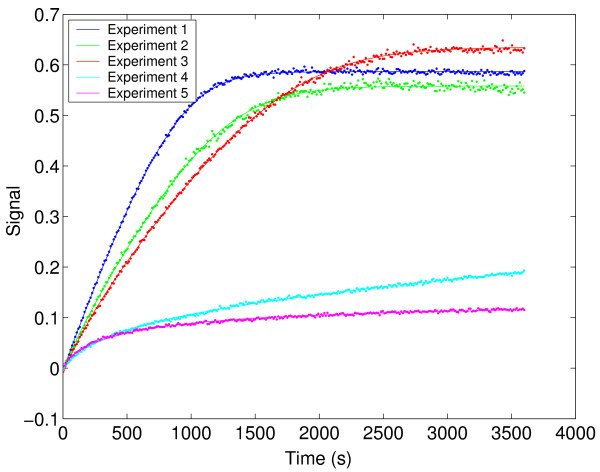
Experimental data versus model prediction for the HIV proteinase case study.

**Figure 11:**
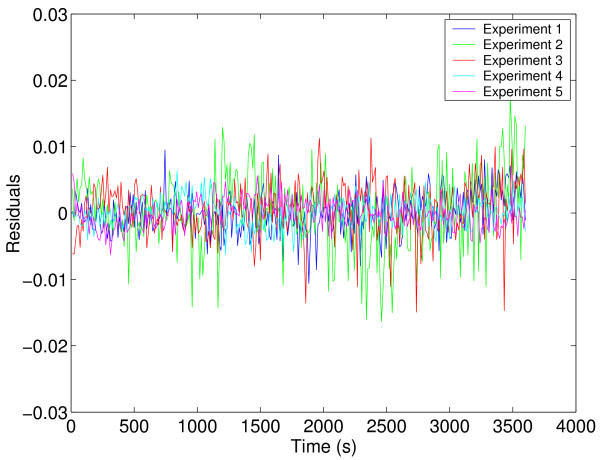
Residuals for the HIV proteinase case study.

However, it is worth noting the very good correlation between the experimental and predicted data for the best decision vector and the lack of correlation between the residuals and time (see Figure 10 and Figure 11).

### Three-step biochemical pathway

**Figure 12:**
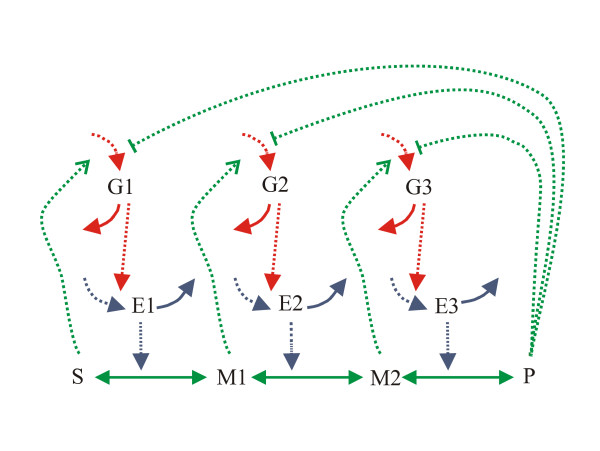
**Three-step biochemical pathway scheme**. The pathway substrate (S) and the product (P) are held at constant concentrations; Ml and M2 are intermediate metabolites of the pathway; El, E2, and E3 are the enzymes and G1, G2, and G3 are the mRNA species for the enzymes. Solid arrows indicate mass transfer reactions and point to the positive direction of flux but are chemical reversible. Dashed arrows indicate activation and dashed curves with blunt ends indicate inhibition.

This case study, considered as a challenging becnhmark problem by Moles and coworkers [19], involves a biochemical pathway with three enzymatic steps, including the enzymes and mRNAs explicitly. Figure 12 contains a diagram illustrating the network of reactions and kinetics effects (feedback loops).

**Figure 13:**
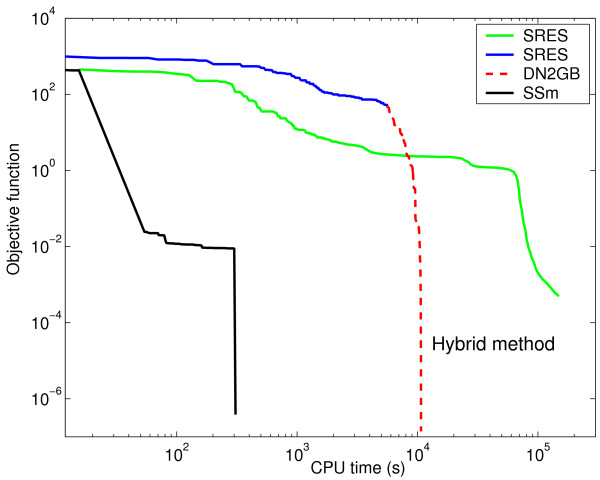
**Convergence curves for the three-step biochemical pathway case study**. Value of cost function versus computation time (in log scale) for SSm, SRES and two-phase hybrid method formed by SRES+DN2FB.

The identification problem consists of the estimation of 36 kinetic parameters of the nonlinear biochemical dynamic model (8 nonlinear ODEs) which describes the variation of the metabolite concentration with time. Moles and coworkers tried to solve this problem using several deterministic and stochastic global optimization algorithms. They found that only a certain type of stochastic algorithms, evolution strategies (implemented as the SRES code), was able to successfully solve it, although at a rather large computational cost. In Figure 13 we can see how the two-phase hybrid method recently presented by Rodriguez-Fernandez and coworkers [13] converged to better solutions, with speeds up of more than one order of magnitude with respect to the previous results.

The novel metaheuristic, SSm, presented in this work was able to improve this result in an additional order of magnitude regarding the computational time. Moreover, SSm had the additional advantage of not requiring preliminary runs, or any user inputs, for tuning the method, making it a very easy to use strategy. In short, using SSm we have reduced the computation time from two days [19] to a couple of minutes, while ensuring robustness.

**Figure 14:**
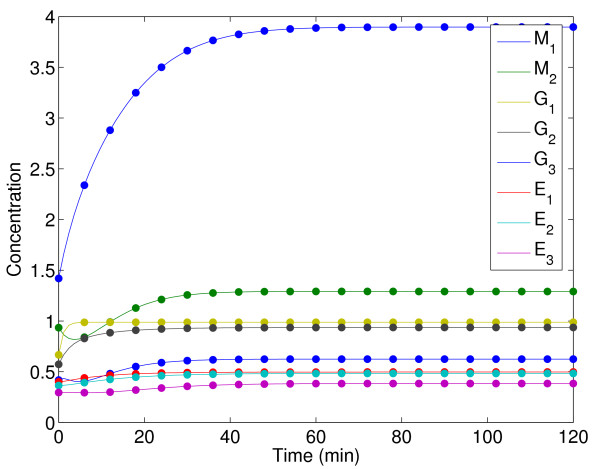
Experimental data versus model prediction for the three-step biochemical pathway case study.

**Figure 15:**
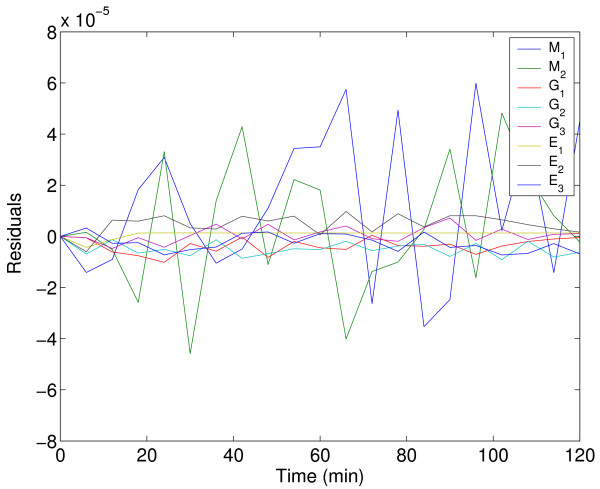
Residuals for the three-step biochemical pathway case study.

Figure 14 shows a comparison (between the predicted and experimental data) for one of the experiments evidencing the accuracy of the fit. Figure 15 confirms that the residuals are white. The representation of the dynamic behavior for the other experiments is quite similar and is not included here for the sake of brevity.

**Figure 16:**
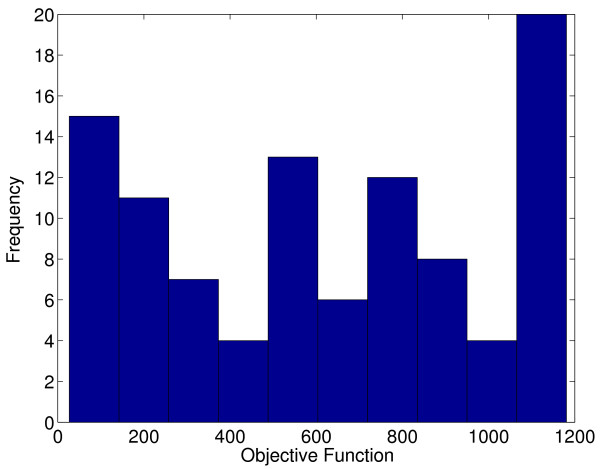
**Multistart for the three-step biochemical pathway case study**. Frequency of the solutions for a multistart using N2FB.

It is sometimes argued that a multistart local method can solve almost all global optimization problems. This can be false for even small problems [54]. The histogram in Figure 16 represents the frequency of the solutions for a multistart of 100 runs using N2FB. The global optimum is in this region close to zero but we can see that it was never reached while a very large number of solutions are far from the global optimum. Despite the identifiability difficulties of this problem, which make most of the solvers fail when trying to solve it, the confidence intervals of the global solution are small indicating a precise parameter estimation. This fact is discussed in more detail in [13].

## Conclusion

Parameter estimation from experimental data remains a bottleneck for a major breakthrough in systems biology. Traditional global optimization methods can ensure proper solutions, but suffer from the large computational burden required for large-scale model identification. In this contribution, we have presented a novel global optimization metaheuristic, SSm, which increases very significantly the efficiency of the estimation while keeping robustness. Its capabilities were tested considering three challenging benchmark problems. This new method was able to successfully find the best known solutions for these problems while reducing the computation time by several orders of magnitude with respect to previous approaches.

## Methods

### Problem statement

In this work, we consider deterministic, nonlinear dynamic models of biochemical systems, i.e. those described by deterministic ordinary differential equations (ODEs), or differential-algebraic equations (DAEs). In the case of ODEs, a popular statement is the so called state-space formulation:

*x*(*p,t*) = *f*[*x*(*p,t*), *u*(*t*),*p*], *x*(0) = x_0_,     (8)

*y*(*p,t*) = *g*[*x*(*p,t*), *u*(*p,t*), *p*]     (9)

where *x *is the vector of *N*_*x *_state variables and *p *the vector of *N*_*p *_model parameters. Note that *f *specify the model, *u *specifies the vector of inputs (i.e. for a particular experiment) and *y *the vector of *N*_*y *_measured states. An experiment is specified by the initial conditions *x*(0), the inputs *u *chosen from among some set of possible inputs *U *and the observations *y*. Note that the inputs can be time dependent.

Given a model structure and a set of experimental data, the goal of the parameter estimation problem is to calibrate the model so as to reproduce the experimental results in the best possible way. This is performed by minimizing a cost function that measures the goodness of the fit. Several estimator functions have been suggested as metrics, standing out the maximum likelihood estimator introduced by Fisher (1912), for being the one that maximizes the probability of the observed event.

Maximum likelihood estimation consists of maximizing the so-called likelihood function, *J*_*ml *_, which is the probability density of a model for the occurrence of the measurements for given parameters. The likelihood function depends on the probability of the measurements. Assuming these to be uncorrelated normal distributions, the log-likelihood function (which yields the same estimate that the likelihood function but is easier to handle in practice) is given as:

Jml(p)=N2ln(2π)+12∑i=1N[ln(σi2)+(y˜i−yi(p))2σi2]     (10)
 MathType@MTEF@5@5@+=feaafiart1ev1aaatCvAUfKttLearuWrP9MDH5MBPbIqV92AaeXatLxBI9gBaebbnrfifHhDYfgasaacH8akY=wiFfYdH8Gipec8Eeeu0xXdbba9frFj0=OqFfea0dXdd9vqai=hGuQ8kuc9pgc9s8qqaq=dirpe0xb9q8qiLsFr0=vr0=vr0dc8meaabaqaciaacaGaaeqabaqabeGadaaakeaacqWGkbGsdaWgaaWcbaGaemyBa0MaemiBaWgabeaakiabcIcaOiabdchaWjabcMcaPiabg2da9maalaaabaGaemOta4eabaGaeGOmaidaaGqaciab=XgaSjab=5gaUjabcIcaOiabikdaYGGaciab+b8aWjabcMcaPiabgUcaRmaalaaabaGaeGymaedabaGaeGOmaidaamaaqahabaWaamWaaeaacqWFSbaBcqWFUbGBcqGGOaakcqGFdpWCdaqhaaWcbaGaemyAaKgabaGaeGOmaidaaOGaeiykaKIaey4kaSYaaSaaaeaacqGGOaakcuWG5bqEgaacamaaBaaaleaacqWGPbqAaeqaaOGaeyOeI0IaemyEaK3aaSbaaSqaaiabdMgaPbqabaGccqGGOaakcqWGWbaCcqGGPaqkcqGGPaqkdaahaaWcbeqaaiabikdaYaaaaOqaaiab+n8aZnaaDaaaleaacqWGPbqAaeaacqaIYaGmaaaaaaGccaGLBbGaayzxaaaaleaacqWGPbqAcqGH9aqpcqaIXaqmaeaacqWGobGta0GaeyyeIuoakiaaxMaacaWLjaWaaeWaaeaacqaIXaqmcqaIWaamaiaawIcacaGLPaaaaaa@695C@

For given measurements y˜i
 MathType@MTEF@5@5@+=feaafiart1ev1aaatCvAUfKttLearuWrP9MDH5MBPbIqV92AaeXatLxBI9gBaebbnrfifHhDYfgasaacH8akY=wiFfYdH8Gipec8Eeeu0xXdbba9frFj0=OqFfea0dXdd9vqai=hGuQ8kuc9pgc9s8qqaq=dirpe0xb9q8qiLsFr0=vr0=vr0dc8meaabaqaciaacaGaaeqabaqabeGadaaakeaacuWG5bqEgaacamaaBaaaleaacqWGPbqAaeqaaaaa@2FBD@, the maximum likelihood estimates of the parameters are those values of *p *for which the likelihood function has its minimum. Moreover, if we assume the noise to be Gaussian with known of constant (homoscedastic) variance, minimizing *J*_*ml *_(Equation 10) is equivalent to minimizing the function:

*J*_*ls*_(*p*) = *w*_*i*_[y˜i
 MathType@MTEF@5@5@+=feaafiart1ev1aaatCvAUfKttLearuWrP9MDH5MBPbIqV92AaeXatLxBI9gBaebbnrfifHhDYfgasaacH8akY=wiFfYdH8Gipec8Eeeu0xXdbba9frFj0=OqFfea0dXdd9vqai=hGuQ8kuc9pgc9s8qqaq=dirpe0xb9q8qiLsFr0=vr0=vr0dc8meaabaqaciaacaGaaeqabaqabeGadaaakeaacuWG5bqEgaacamaaBaaaleaacqWGPbqAaeqaaaaa@2FBD@ - *y*_*i*_(*p*)]^2 ^    (11)

with the weights *W*_*i *_= 1σ2
 MathType@MTEF@5@5@+=feaafiart1ev1aaatCvAUfKttLearuWrP9MDH5MBPbIqV92AaeXatLxBI9gBaebbnrfifHhDYfgasaacH8akY=wiFfYdH8Gipec8Eeeu0xXdbba9frFj0=OqFfea0dXdd9vqai=hGuQ8kuc9pgc9s8qqaq=dirpe0xb9q8qiLsFr0=vr0=vr0dc8meaabaqaciaacaGaaeqabaqabeGadaaakeaadaWcaaqaaiabigdaXaqaaGGaciab=n8aZnaaCaaaleqabaGaeGOmaidaaaaaaaa@3095@. One thus obtains a weighted least-squares estimator. If all *σ*_*i*_'s are equal, unweighed least-squares should be used (*w*_*i *_= 1) and the noise variance do not need to be known a priori and can be estimated a posteriori from the residuals [7,8].

### Confidence intervals

In general, confidence regions can be expressed as:

{p:(p−p^)TC−1(p−p^)T≤npFnp,N−np1−α}     (12)
 MathType@MTEF@5@5@+=feaafiart1ev1aaatCvAUfKttLearuWrP9MDH5MBPbIqV92AaeXatLxBI9gBaebbnrfifHhDYfgasaacH8akY=wiFfYdH8Gipec8Eeeu0xXdbba9frFj0=OqFfea0dXdd9vqai=hGuQ8kuc9pgc9s8qqaq=dirpe0xb9q8qiLsFr0=vr0=vr0dc8meaabaqaciaacaGaaeqabaqabeGadaaakeaacqGG7bWEcqWGWbaCcqGG6aGocqGGOaakcqWGWbaCcqGHsislcuWGWbaCgaqcaiabcMcaPmaaCaaaleqabaGaemivaqfaaOGaem4qam0aaWbaaSqabeaacqGHsislcqaIXaqmaaGccqGGOaakcqWGWbaCcqGHsislcuWGWbaCgaqcaiabcMcaPmaaCaaaleqabaGaemivaqfaaOGaeyizImQaemOBa42aaSbaaSqaaiabdchaWbqabaGccqWGgbGrdaqhaaWcbaGaemOBa42aaSbaaWqaaiabdchaWbqabaWccqGGSaalcqWGobGtcqGHsislcqWGUbGBdaWgaaadbaGaemiCaahabeaaaSqaaiabigdaXiabgkHiTGGaciab=f7aHbaakiabc2ha9jaaxMaacaWLjaWaaeWaaeaacqaIXaqmcqaIYaGmaiaawIcacaGLPaaaaaa@5A3B@

The covariance matrix obtained for a linear case can be extended for nonlinear models leading an approximate covariance matrix as:

CJ(p^)=J(p^)N−np[∑i=1N(∂yi∂p(p^))TV−1(∂yi∂p(p^))]−1     (13)
 MathType@MTEF@5@5@+=feaafiart1ev1aaatCvAUfKttLearuWrP9MDH5MBPbIqV92AaeXatLxBI9gBaebbnrfifHhDYfgasaacH8akY=wiFfYdH8Gipec8Eeeu0xXdbba9frFj0=OqFfea0dXdd9vqai=hGuQ8kuc9pgc9s8qqaq=dirpe0xb9q8qiLsFr0=vr0=vr0dc8meaabaqaciaacaGaaeqabaqabeGadaaakeaacqWGdbWqdaWgaaWcbaGaemOsaOeabeaakiabcIcaOiqbdchaWzaajaGaeiykaKIaeyypa0ZaaSaaaeaacqWGkbGscqGGOaakcuWGWbaCgaqcaiabcMcaPaqaaiabd6eaojabgkHiTiabd6gaUnaaBaaaleaacqWGWbaCaeqaaaaakmaadmaabaWaaabCaeaadaqadaqaamaalaaabaGaeyOaIyRaemyEaK3aaSbaaSqaaiabdMgaPbqabaaakeaacqGHciITcqWGWbaCaaGaeiikaGIafmiCaaNbaKaacqGGPaqkaiaawIcacaGLPaaaaSqaaiabdMgaPjabg2da9iabigdaXaqaaiabd6eaobqdcqGHris5aOWaaWbaaSqabeaacqWGubavaaGccqWGwbGvdaahaaWcbeqaaiabgkHiTiabigdaXaaakmaabmaabaWaaSaaaeaacqGHciITcqWG5bqEdaWgaaWcbaGaemyAaKgabeaaaOqaaiabgkGi2kabdchaWbaacqGGOaakcuWGWbaCgaqcaiabcMcaPaGaayjkaiaawMcaaaGaay5waiaaw2faamaaCaaaleqabaGaeyOeI0IaeGymaedaaOGaaCzcaiaaxMaadaqadaqaaiabigdaXiabiodaZaGaayjkaiaawMcaaaaa@68E1@

where the term J(p^)N−np
 MathType@MTEF@5@5@+=feaafiart1ev1aaatCvAUfKttLearuWrP9MDH5MBPbIqV92AaeXatLxBI9gBaebbnrfifHhDYfgasaacH8akY=wiFfYdH8Gipec8Eeeu0xXdbba9frFj0=OqFfea0dXdd9vqai=hGuQ8kuc9pgc9s8qqaq=dirpe0xb9q8qiLsFr0=vr0=vr0dc8meaabaqaciaacaGaaeqabaqabeGadaaakeaadaWcaaqaaiabdQeakjabcIcaOiqbdchaWzaajaGaeiykaKcabaGaemOta4KaeyOeI0IaemOBa42aaSbaaSqaaiabdchaWbqabaaaaaaa@3610@ is an unbiased approximation of the residual variance *σ*^2 ^and ∂y∂p(p^)
 MathType@MTEF@5@5@+=feaafiart1ev1aaatCvAUfKttLearuWrP9MDH5MBPbIqV92AaeXatLxBI9gBaebbnrfifHhDYfgasaacH8akY=wiFfYdH8Gipec8Eeeu0xXdbba9frFj0=OqFfea0dXdd9vqai=hGuQ8kuc9pgc9s8qqaq=dirpe0xb9q8qiLsFr0=vr0=vr0dc8meaabaqaciaacaGaaeqabaqabeGadaaakeaadaWcaaqaaiabgkGi2kabdMha5bqaaiabgkGi2kabdchaWbaacqGGOaakcuWGWbaCgaqcaiabcMcaPaaa@3597@ the sensitivity functions with respect to the parameters evaluated at p^
 MathType@MTEF@5@5@+=feaafiart1ev1aaatCvAUfKttLearuWrP9MDH5MBPbIqV92AaeXatLxBI9gBaebbnrfifHhDYfgasaacH8akY=wiFfYdH8Gipec8Eeeu0xXdbba9frFj0=OqFfea0dXdd9vqai=hGuQ8kuc9pgc9s8qqaq=dirpe0xb9q8qiLsFr0=vr0=vr0dc8meaabaqaciaacaGaaeqabaqabeGadaaakeaacuWGWbaCgaqcaaaa@2E25@.

Under the assumption of uncorrelated measurement noise with Gaussian distribution with a mean of zero, the approximation of the covariance matrix *C*_*J *_given by the Equation 13 is just the inverse of the Fisher information matrix of the estimation problem defined as:

FIM(p^)=∑i=1N(∂yi∂p(p^))TV−2(∂yi∂p(p^))     (14)
 MathType@MTEF@5@5@+=feaafiart1ev1aaatCvAUfKttLearuWrP9MDH5MBPbIqV92AaeXatLxBI9gBaebbnrfifHhDYfgasaacH8akY=wiFfYdH8Gipec8Eeeu0xXdbba9frFj0=OqFfea0dXdd9vqai=hGuQ8kuc9pgc9s8qqaq=dirpe0xb9q8qiLsFr0=vr0=vr0dc8meaabaqaciaacaGaaeqabaqabeGadaaakeaacqWGgbGrcqWGjbqscqWGnbqtcqGGOaakcuWGWbaCgaqcaiabcMcaPiabg2da9maaqahabaWaaeWaaeaadaWcaaqaaiabgkGi2kabdMha5naaBaaaleaacqWGPbqAaeqaaaGcbaGaeyOaIyRaemiCaahaaiabcIcaOiqbdchaWzaajaGaeiykaKcacaGLOaGaayzkaaWaaWbaaSqabeaacqWGubavaaaabaGaemyAaKMaeyypa0JaeGymaedabaGaemOta4eaniabggHiLdGccqWGwbGvdaahaaWcbeqaaiabgkHiTiabikdaYaaakmaabmaabaWaaSaaaeaacqGHciITcqWG5bqEdaWgaaWcbaGaemyAaKgabeaaaOqaaiabgkGi2kabdchaWbaacqGGOaakcuWGWbaCgaqcaiabcMcaPaGaayjkaiaawMcaaiaaxMaacaWLjaWaaeWaaeaacqaIXaqmcqaI0aanaiaawIcacaGLPaaaaaa@5C4D@

According to the Cramèr-Rao theorem, *C*_*J*_(p^
 MathType@MTEF@5@5@+=feaafiart1ev1aaatCvAUfKttLearuWrP9MDH5MBPbIqV92AaeXatLxBI9gBaebbnrfifHhDYfgasaacH8akY=wiFfYdH8Gipec8Eeeu0xXdbba9frFj0=OqFfea0dXdd9vqai=hGuQ8kuc9pgc9s8qqaq=dirpe0xb9q8qiLsFr0=vr0=vr0dc8meaabaqaciaacaGaaeqabaqabeGadaaakeaacuWGWbaCgaqcaaaa@2E25@) = *FIM*^-1 ^represents the error covariance matrix of the minimum variance unbiased estimator, thus substituting *C*_*J *_from Equation 13 into Equation 12, yields the approximate confidence ellipsoids.

Therefore, a lower bound for the individual parameter confidence interval *σ*_*i *_(*i *= 1,...,*n*_*p*_) can be obtained from the diagonal of the covariance matrix as:

δi=±tN−np1−(α/2)Cii     (15)
 MathType@MTEF@5@5@+=feaafiart1ev1aaatCvAUfKttLearuWrP9MDH5MBPbIqV92AaeXatLxBI9gBaebbnrfifHhDYfgasaacH8akY=wiFfYdH8Gipec8Eeeu0xXdbba9frFj0=OqFfea0dXdd9vqai=hGuQ8kuc9pgc9s8qqaq=dirpe0xb9q8qiLsFr0=vr0=vr0dc8meaabaqaciaacaGaaeqabaqabeGadaaakeaaiiGacqWF0oazdaWgaaWcbaGaemyAaKgabeaakiabg2da9iabgglaXkabdsha0naaDaaaleaacqWGobGtcqGHsislcqWGUbGBdaWgaaadbaGaemiCaahabeaaaSqaaiabigdaXiabgkHiTiabcIcaOiab=f7aHjabc+caViabikdaYiabcMcaPaaakmaakaaabaGaem4qam0aaSbaaSqaaiabdMgaPjabdMgaPbqabaaabeaakiaaxMaacaWLjaWaaeWaaeaacqaIXaqmcqaI1aqnaiaawIcacaGLPaaaaaa@495E@

where tN−np1−(α/2)
 MathType@MTEF@5@5@+=feaafiart1ev1aaatCvAUfKttLearuWrP9MDH5MBPbIqV92AaeXatLxBI9gBaebbnrfifHhDYfgasaacH8akY=wiFfYdH8Gipec8Eeeu0xXdbba9frFj0=OqFfea0dXdd9vqai=hGuQ8kuc9pgc9s8qqaq=dirpe0xb9q8qiLsFr0=vr0=vr0dc8meaabaqaciaacaGaaeqabaqabeGadaaakeaacqWG0baDdaqhaaWcbaGaemOta4KaeyOeI0IaemOBa42aaSbaaWqaaiabdchaWbqabaaaleaacqaIXaqmcqGHsislcqGGOaakiiGacqWFXoqycqGGVaWlcqaIYaGmcqGGPaqkaaaaaa@3A6F@ is the two-tails Student's *t *distribution for the given confidence level *α *and *N *- *n*_*p *_degrees of freedom which converges to a linear distribution when the number of measurements *N *is high. Assuming that the measurement noise is white and uncorrelated we consider the error correlation matrix as diagonal, neglecting the off-diagonal elements of *C*, that is, the covariances among the parameters. When parameters are simultaneously determined they usually have a significant covariance thus the confidence intervals might be underestimated. That is why these confidence intervals obtained from the FIM can only be taken as lower bounds and never as an exact confidence region.

### A posteriori local identifiability analysis

Under the assumption of uncorrelated measurement noise with Gaussian distribution with a mean of zero, the covariance matrix can be approximated by the inverse of the Fisher information matrix involving the output sensitivity functions. If the sensitivity equations shows linear dependence at the experimental data points, the FIM becomes singular and the model is not identifiable.

Useful information about the correlation between estimated parameters can be also obtained from the covariance matrix. The correlation matrix, which elements are the approximate correlation coefficients between the i-th and the j-th parameter, is defined by:

Rij=CijCiiCjj,i≠j,     (16)
 MathType@MTEF@5@5@+=feaafiart1ev1aaatCvAUfKttLearuWrP9MDH5MBPbIqV92AaeXatLxBI9gBaebbnrfifHhDYfgasaacH8akY=wiFfYdH8Gipec8Eeeu0xXdbba9frFj0=OqFfea0dXdd9vqai=hGuQ8kuc9pgc9s8qqaq=dirpe0xb9q8qiLsFr0=vr0=vr0dc8meaabaqaciaacaGaaeqabaqabeGadaaakeaacqWGsbGudaWgaaWcbaGaemyAaKMaemOAaOgabeaakiabg2da9maalaaabaGaem4qam0aaSbaaSqaaiabdMgaPjabdQgaQbqabaaakeaadaGcaaqaaiabdoeadnaaBaaaleaacqWGPbqAcqWGPbqAaeqaaOGaem4qam0aaSbaaSqaaiabdQgaQjabdQgaQbqabaaabeaaaaGccqGGSaalcqWGPbqAcqGHGjsUcqWGQbGAcqGGSaalcaWLjaGaaCzcamaabmaabaGaeGymaeJaeGOnaydacaGLOaGaayzkaaaaaa@48DA@

*R*_*ij *_= 1, *i *= *j*,     (17)

A singular FIM indicates the presence of unidentifiable parameters, and correlations between parameters that are greater than 0.99 may lead to singular FIM.

### SSm algorithm

SSm is an advanced design of the scatter search algorithm for real variables. The method uses a relatively short number, *b*, of *elite *decision vectors in a so-called *reference set *(*refset*). These *elite *vectors are combined in pairs to generate new ones that may enter the *refset *replacing existing vectors in it (i.e. the *refset *always maintains a fixed number of vectors). This evolutionary approach is combined with local searches from selected vectors.

In a n-dimensional problem, vectors of decision variables are represented by, *x *∈ ℝ^*n *^so that a particular decision variable in the population of size NP can be symbolized as xir
 MathType@MTEF@5@5@+=feaafiart1ev1aaatCvAUfKttLearuWrP9MDH5MBPbIqV92AaeXatLxBI9gBaebbnrfifHhDYfgasaacH8akY=wiFfYdH8Gipec8Eeeu0xXdbba9frFj0=OqFfea0dXdd9vqai=hGuQ8kuc9pgc9s8qqaq=dirpe0xb9q8qiLsFr0=vr0=vr0dc8meaabaqaciaacaGaaeqabaqabeGadaaakeaacqWG4baEdaqhaaWcbaGaemyAaKgabaGaemOCaihaaaaa@311A@, where *i *= 1, 2,...,*n *and *r *= 1, 2,...,*NP*. The *refset *will have *NP *= *b*, whose default value is 10.

The main steps of the algorithm are shown below, with a diagram presented in Figure 1.

#### Generation of diverse vectors within the search space

The first step consists of generating a set *S *of *m *(default *m *= 10 · *b*) diverse vectors in the search space. Unlike other diversification strategies, SSm does not only generate vectors with their components uniformly distributed within the search space, but also drives the generation of values for each decision variable onto parts of the space where they have not appeared very often during the search process. For that, the method makes use of memory taking into account the number of times that every decision variable appears in different parts of the search space.

Initially, the range of every decision variable, defined by its lower and upper bounds, *xl*_*i *_and *xu*_*i *_respectively, is divided in *p *(default *p *= 4) subranges of equal size, (*xu*_*i *_- *xl*_*i*_)/*p*. Therefore, the limits that define each subrange *j *∈ [1, 2,...,*p*] for the variable *i *are given by

• Lower bound:

lbij=xli+xui−xlip(j−1)     (18)
 MathType@MTEF@5@5@+=feaafiart1ev1aaatCvAUfKttLearuWrP9MDH5MBPbIqV92AaeXatLxBI9gBaebbnrfifHhDYfgasaacH8akY=wiFfYdH8Gipec8Eeeu0xXdbba9frFj0=OqFfea0dXdd9vqai=hGuQ8kuc9pgc9s8qqaq=dirpe0xb9q8qiLsFr0=vr0=vr0dc8meaabaqaciaacaGaaeqabaqabeGadaaakeaacqWGSbaBcqWGIbGydaWgaaWcbaGaemyAaKMaemOAaOgabeaakiabg2da9iabdIha4jabdYgaSnaaBaaaleaacqWGPbqAaeqaaOGaey4kaSYaaSaaaeaacqWG4baEcqWG1bqDdaWgaaWcbaGaemyAaKgabeaakiabgkHiTiabdIha4jabdYgaSnaaBaaaleaacqWGPbqAaeqaaaGcbaGaemiCaahaaiabcIcaOiabdQgaQjabgkHiTiabigdaXiabcMcaPiaaxMaacaWLjaWaaeWaaeaacqaIXaqmcqaI4aaoaiaawIcacaGLPaaaaaa@4D90@

• Upper bound:

ubij=xli+xui−xlipj     (19)
 MathType@MTEF@5@5@+=feaafiart1ev1aaatCvAUfKttLearuWrP9MDH5MBPbIqV92AaeXatLxBI9gBaebbnrfifHhDYfgasaacH8akY=wiFfYdH8Gipec8Eeeu0xXdbba9frFj0=OqFfea0dXdd9vqai=hGuQ8kuc9pgc9s8qqaq=dirpe0xb9q8qiLsFr0=vr0=vr0dc8meaabaqaciaacaGaaeqabaqabeGadaaakeaacqWG1bqDcqWGIbGydaWgaaWcbaGaemyAaKMaemOAaOgabeaakiabg2da9iabdIha4jabdYgaSnaaBaaaleaacqWGPbqAaeqaaOGaey4kaSYaaSaaaeaacqWG4baEcqWG1bqDdaWgaaWcbaGaemyAaKgabeaakiabgkHiTiabdIha4jabdYgaSnaaBaaaleaacqWGPbqAaeqaaaGcbaGaemiCaahaaiabdQgaQjaaxMaacaWLjaWaaeWaaeaacqaIXaqmcqaI5aqoaiaawIcacaGLPaaaaaa@4A15@

Frequencies *f*_*ij *_are defined as the number of times that the variable *i *is in the sub range *j *along all the generated vectors, with *i *∈ [1,2,...,*n*] and *j *∈ [1,2,...,*p*].

To initialize all the frequencies to a value of 1, *p *vectors are first generated, each of them having all their variables randomly generated in the same sub range using a uniform distribution (e.g. vector 1, *x*^1^, has all its variables in sub range 1, and every decision variable i is randomly generated using a uniform distribution within the bounds *xl*_*i *_and xli+(xui−xli)p
 MathType@MTEF@5@5@+=feaafiart1ev1aaatCvAUfKttLearuWrP9MDH5MBPbIqV92AaeXatLxBI9gBaebbnrfifHhDYfgasaacH8akY=wiFfYdH8Gipec8Eeeu0xXdbba9frFj0=OqFfea0dXdd9vqai=hGuQ8kuc9pgc9s8qqaq=dirpe0xb9q8qiLsFr0=vr0=vr0dc8meaabaqaciaacaGaaeqabaqabeGadaaakeaacqWG4baEcqWGSbaBdaWgaaWcbaGaemyAaKgabeaakiabgUcaRmaalaaabaGaeiikaGIaemiEaGNaemyDau3aaSbaaSqaaiabdMgaPbqabaGccqGHsislcqWG4baEcqWGSbaBdaWgaaWcbaGaemyAaKgabeaakiabcMcaPaqaaiabdchaWbaaaaa@3EF9@). This first set of vectors forms the initial matrix of diverse vectors *S*^*p *× *n *^that will be extended up to a size of *S*^*m *× *n *^by adding new diverse vectors.

S=[x1x2⋮xp] with f=(f11f12⋯f1pf21f22⋯f2p⋮⋮⋮⋮fn1fn2⋯fnp)=ones(n,p)     (20)
 MathType@MTEF@5@5@+=feaafiart1ev1aaatCvAUfKttLearuWrP9MDH5MBPbIqV92AaeXatLxBI9gBaebbnrfifHhDYfgasaacH8akY=wiFfYdH8Gipec8Eeeu0xXdbba9frFj0=OqFfea0dXdd9vqai=hGuQ8kuc9pgc9s8qqaq=dirpe0xb9q8qiLsFr0=vr0=vr0dc8meaabaqaciaacaGaaeqabaqabeGadaaakeaacqWGtbWucqGH9aqpdaWadaqaauaabeqaeeaaaaqaaiabdIha4naaCaaaleqabaGaeGymaedaaaGcbaGaemiEaG3aaWbaaSqabeaacqaIYaGmaaaakeaacqWIUlstaeaacqWG4baEdaahaaWcbeqaaiabdchaWbaaaaaakiaawUfacaGLDbaacqqGGaaicqWG3bWDcqWGPbqAcqWG0baDcqWGObaAcqqGGaaicqWGMbGzcqGH9aqpdaqadaqaauaabeqaeqaaaaaabaGaemOzay2aaSbaaSqaaiabigdaXiabigdaXaqabaaakeaacqWGMbGzdaWgaaWcbaGaeGymaeJaeGOmaidabeaaaOqaaiabl+UimbqaaiabdAgaMnaaBaaaleaacqaIXaqmcqWGWbaCaeqaaaGcbaGaemOzay2aaSbaaSqaaiabikdaYiabigdaXaqabaaakeaacqWGMbGzdaWgaaWcbaGaeGOmaiJaeGOmaidabeaaaOqaaiabl+UimbqaaiabdAgaMnaaBaaaleaacqaIYaGmcqWGWbaCaeqaaaGcbaGaeSO7I0eabaGaeSO7I0eabaGaeSO7I0eabaGaeSO7I0eabaGaemOzay2aaSbaaSqaaiabd6gaUjabigdaXaqabaaakeaacqWGMbGzdaWgaaWcbaGaemOBa4MaeGOmaidabeaaaOqaaiabl+UimbqaaiabdAgaMnaaBaaaleaacqWGUbGBcqWGWbaCaeqaaaaaaOGaayjkaiaawMcaaiabg2da9iabd+gaVjabd6gaUjabdwgaLjabdohaZjabcIcaOiabd6gaUjabcYcaSiabdchaWjabcMcaPiaaxMaacaWLjaWaaeWaaeaacqaIYaGmcqaIWaamaiaawIcacaGLPaaaaaa@8617@

New vectors will be generated using the following procedure:

For each new vector *x*^*p*+*t *^to be generated, the probability of having its decision variable *i *in the sub range *j *is calculated as

probi,jp+t=1fij∑k=1p1fik     (21)
 MathType@MTEF@5@5@+=feaafiart1ev1aaatCvAUfKttLearuWrP9MDH5MBPbIqV92AaeXatLxBI9gBaebbnrfifHhDYfgasaacH8akY=wiFfYdH8Gipec8Eeeu0xXdbba9frFj0=OqFfea0dXdd9vqai=hGuQ8kuc9pgc9s8qqaq=dirpe0xb9q8qiLsFr0=vr0=vr0dc8meaabaqaciaacaGaaeqabaqabeGadaaakeaacqWGWbaCcqWGYbGCcqWGVbWBcqWGIbGydaqhaaWcbaGaemyAaKMaeiilaWIaemOAaOgabaGaemiCaaNaey4kaSIaemiDaqhaaOGaeyypa0ZaaSaaaeaadaWcaaqaaiabigdaXaqaaiabdAgaMnaaBaaaleaacqWGPbqAcqWGQbGAaeqaaaaaaOqaamaaqadabaWaaSaaaeaacqaIXaqmaeaacqWGMbGzdaWgaaWcbaGaemyAaKMaem4AaSgabeaaaaaabaGaem4AaSMaeyypa0JaeGymaedabaGaemiCaahaniabggHiLdaaaOGaaCzcaiaaxMaadaqadaqaaiabikdaYiabigdaXaGaayjkaiaawMcaaaaa@50C3@

with *t *∈ [1,2,...,*m *- *p*], *i *∈ [1,2,...,*n*] and *j *∈ [1,2,...,*p*].

Then, a uniformly distributed random number, *rnd*, in the interval [0 1] is generated. The next generated vector *x*^*p*+*t *^will have its i-th component in the subrange *j *= *a *for the first value of *a *that accomplishes

rnd≤∑j=1aprobi,jp+t a=1,2,...,p     (22)
 MathType@MTEF@5@5@+=feaafiart1ev1aaatCvAUfKttLearuWrP9MDH5MBPbIqV92AaeXatLxBI9gBaebbnrfifHhDYfgasaacH8akY=wiFfYdH8Gipec8Eeeu0xXdbba9frFj0=OqFfea0dXdd9vqai=hGuQ8kuc9pgc9s8qqaq=dirpe0xb9q8qiLsFr0=vr0=vr0dc8meaabaqaciaacaGaaeqabaqabeGadaaakeaacqWGYbGCcqWGUbGBcqWGKbazcqGHKjYOdaaeWbqaaiabdchaWjabdkhaYjabd+gaVjabdkgaInaaDaaaleaacqWGPbqAcqGGSaalcqWGQbGAaeaacqWGWbaCcqGHRaWkcqWG0baDaaaabaGaemOAaOMaeyypa0JaeGymaedabaGaemyyaeganiabggHiLdGccqqGGaaicqWGHbqycqGH9aqpcqaIXaqmcqGGSaalcqaIYaGmcqGGSaalcqGGUaGlcqGGUaGlcqGGUaGlcqGGSaalcqWGWbaCcaWLjaGaaCzcamaabmaabaGaeGOmaiJaeGOmaidacaGLOaGaayzkaaaaaa@56CE@

Each component, xip+t
 MathType@MTEF@5@5@+=feaafiart1ev1aaatCvAUfKttLearuWrP9MDH5MBPbIqV92AaeXatLxBI9gBaebbnrfifHhDYfgasaacH8akY=wiFfYdH8Gipec8Eeeu0xXdbba9frFj0=OqFfea0dXdd9vqai=hGuQ8kuc9pgc9s8qqaq=dirpe0xb9q8qiLsFr0=vr0=vr0dc8meaabaqaciaacaGaaeqabaqabeGadaaakeaacqWG4baEdaqhaaWcbaGaemyAaKgabaGaemiCaaNaey4kaSIaemiDaqhaaaaa@3369@, will take a value randomly selected using an uniform distribution in the range [*lb*_*ij*_, *ub*_*ij*_].

Thus, for a new vector to be generated, the probability of having the variable *i *in the subrange *j *is inversely proportional to the frequency of appearance of the variables i in this subrange along the already created vectors. Therefore, the method has to "remember" and update these frequencies to enhance diversity. As new vectors *x*^*p*+*t *^are generated, they are added to the matrix S in rows until it becomes m-by-n dimensional.

#### Refset formation

When the diverse vectors have been generated a selected number of them will create the first *refset*, R. There are two strategies to do it.

The first one consists of evaluating the *fitness f *(*x*) (i.e. the cost function) of all diverse vectors and select the *b*/2 best ones in term of *fitness*. For example in a minimization problem, provided the diverse vectors are sorted according to their *fitness *(the best one first), the initial selection is *R*^*b*/2 × *n *^= [*x*^1^, *x*^2^,...,*x*^*b*/2^] such that

*f*(*x*^*i*^) ≤ *f*(*x*^*j*^) ∀ *j *> *i*, *i *∈ [1,2,...,*b*/2], *j *∈ [2,3,...,*m*]     (23)

The current number of vectors present in the *refset *is computed as *h*. Therefore, in this stage *h *= *b*/2 and the maximum value of *h *is *b*. We complete the *refset *with the remaining diverse vectors not yet included by maximizing the minimum Euclidean distance to the included vectors in the *refset*.

For every diverse vector not yet included in the *refset*, *x*^*d *^with *d *∈ [*h *+ 1, *h *+ 2,...,*m*], Euclidean distances to all current *refset *vectors are computed. The minimum of these distances, *d*_*min*_, is stored for each vector **x**:

*d*_*min*_(*x*^*d*^) = *min*{*d*(*x*^*d*^, *R*}     (24)

where *d*(*x, R*), represents a vector whose components are the Euclidean distances between vector **x **and all the vectors in the matrix *R*.

Then, the vector **x **having the highest minimum distance will join the *refset*, *R *= *R *∪ *x *such that

*d*_*min*_(*x*) = *max*(*d*_*min*_(*x*^*d*^)) ∀ *d *= *h *+ 1, *h *+ 2,...,*m *    (25)

and the value of *h *is increased one unit since a new vector has been added to the *refset*. This is repeated until the *refset *is filled with *b *vectors (i.e. *h *= *b*) so that *R *∈ ℝ^*b *× *n*^.

The second strategy does not take into account the *fitness *of the diverse vectors. The initial *refset *is formed by 3 vectors: one having all the variables in their lower bounds, one having all the variables in their upper bounds and the middle point between these two vectors. This initial *refset R *∈ ℝ^3 × *n *^is completed using the same distance criterion described in the first strategy until it is composed of *b *decision vectors. Please note that the first strategy involves a higher computational cost since the *fitness *of all the diverse vectors has to be evaluated. However, this strategy ensures a better quality of the initial *refset *which can help to converge faster to the global solution.

#### Combination

Unlike genetic algorithms or other evolutionary strategies, scatter search does not use mutation or crossover operators among its members, but combinations among them. SSm combines all the vectors in the *refset *in pairs, making use of memory to avoid the combination of two vectors that have already been combined. The number of vectors created from each pair of *elite *vectors depends on the quality of the latter. These combinations are of the following three types, assuming *x' *and *x" *being the *elite *vectors to be combined and being *x' *superior in quality to *x"*:

• Type 1: *c*_1 _= *x' - d*

• Type 2: *c*_2 _= *x' + d*

• Type 3: *c*_3 _= *x'' *+ *d*

where *d *= *r*.·(*x" *- *x'*)/2

And *r *is a vector of dimension *n *with all its components being uniform random numbers in the interval [0 1].

Please note that the notation.· above indicates that the vectors are multiplied component by component, thus that is not an scalar product.

The vector has the form

d=[d1d2⋮dn]=[r1(x″1−x′1)2r2(x″2−x′2)2⋮rn(x″n−x′n)2]     (26)
 MathType@MTEF@5@5@+=feaafiart1ev1aaatCvAUfKttLearuWrP9MDH5MBPbIqV92AaeXatLxBI9gBaebbnrfifHhDYfgasaacH8akY=wiFfYdH8Gipec8Eeeu0xXdbba9frFj0=OqFfea0dXdd9vqai=hGuQ8kuc9pgc9s8qqaq=dirpe0xb9q8qiLsFr0=vr0=vr0dc8meaabaqaciaacaGaaeqabaqabeGadaaakeaacqWGKbazcqGH9aqpdaWadaqaauaabeqaeeaaaaqaaiabdsgaKnaaBaaaleaacqaIXaqmaeqaaaGcbaGaemizaq2aaSbaaSqaaiabikdaYaqabaaakeaacqWIUlstaeaacqWGKbazdaWgaaWcbaGaemOBa4gabeaaaaaakiaawUfacaGLDbaacqGH9aqpdaWadaqaauaabeqaeeaaaaqaamaalaaabaGaemOCai3aaSbaaSqaaiabigdaXaqabaGccqGGOaakcuWG4baEgaGbamaaBaaaleaacqaIXaqmaeqaaOGaeyOeI0IafmiEaGNbauaadaWgaaWcbaGaeGymaedabeaakiabcMcaPaqaaiabikdaYaaaaeaadaWcaaqaaiabdkhaYnaaBaaaleaacqaIYaGmaeqaaOGaeiikaGIafmiEaGNbayaadaWgaaWcbaGaeGOmaidabeaakiabgkHiTiqbdIha4zaafaWaaSbaaSqaaiabikdaYaqabaGccqGGPaqkaeaacqaIYaGmaaaabaGaeSO7I0eabaWaaSaaaeaacqWGYbGCdaWgaaWcbaGaemOBa4gabeaakiabcIcaOiqbdIha4zaagaWaaSbaaSqaaiabd6gaUbqabaGccqGHsislcuWG4baEgaqbamaaBaaaleaacqWGUbGBaeqaaOGaeiykaKcabaGaeGOmaidaaaaaaiaawUfacaGLDbaacaWLjaGaaCzcamaabmaabaGaeGOmaiJaeGOnaydacaGLOaGaayzkaaaaaa@6886@

If both *x' *and *x" *belong to the best *b*/2 elements of the *refset *in terms of *fitness*, then 4 vectors are generated: one of type 1, one of type 3 and two of type 2.

If only *x' *belongs to the best *b*/2 elements of the *refset *in terms of *fitness*, then 3 vectors are generated: one of each type.

If neither *x" *nor *x' *belong to the best *b*/2 elements of the *refset *in terms of *fitness*, then 2 vectors are generated: one of type 2 and another one of type 1 or 3 (randomly chosen).

This type of combination allows more diversity in the generated vectors than the linear combination used in classical implementations of scatter search. These vectors generated by combination of *refset *members will be named *x*^*c *^with *c *∈ [1, 2,..., *nc*], and form a matrix *C *∈ ℝ^*nc *× *n *^where *nc *is the total number of vectors generated by combination, which is not a fixed number. It may change every iteration depending on the number of combinations made among *refset *members (remember that the method avoids doing combinations with pairs of vectors already combined).

#### Refset update

Once the combinations have been done, the new vectors generated may replace the *elite *vectors if the *refset *can increase its quality. Each new vector created by combination which is better than the worst vector in *refset *is compared with all the *elite *vectors. If new vectors outperform *elite *vectors in terms of *fitness *they replace them as long as they comply with a minimum diversity (i.e. the method avoids vector duplication in the *refset *by computing Euclidean distances among all vectors).

The best generated vector by combination is compared with the worst vector in *refset*. If the former outperforms the latter and is not included in the *refset*, the replacement is carried out. Otherwise, the algorithm tries to find another vector in the *refset *to accomplish both conditions and do the replacement.

The first candidate vector to join the *refset *among the *nc *generated vectors by combination is *z *such that

*f*(*z*) ≤ *f*(*x*^*i*^) ∀ *i *= 1,2,...,*nc *    (27)

The possible vector to be replaced in the *refset *is the worst in the *refset*, *x*^*w *^such that

*f*(*x*^*w*^) ≥ *f*(*x*^*j*^) ∀ *j *= 1,2,...,*b *    (28)

The replacement will be carried out if

*f*(*z*) ≤ *f*(*x*^*w*^) *and z *∉ *R *    (29)

Regardless the replacement is done or not, *z *is deleted from the matrix *C*, therefore *nc *is decreased in one unit. This is repeated with every generated vector by combination until no new vectors are better in quality than the current worst vector in *refset*.

There is an exception to these rules: if one vector has the best *fitness *in terms of quality found so far, it will join the *refset *replacing the worst vector in it or, in case that the diversity condition can not be achieved, the closest *elite *vector to it.

A mechanism to avoid flat zones is added to the *refset *update. In flat areas, many vectors with very similar (and sometimes the same) *fitness *can appear. To avoid including vectors from the same flat area, new vectors can only join the *refset *if the candidate vector has a different *fitness *value apart from being diverse enough. This prevents vectors in the same flat area from joining the *refset *at the same time.

Provided the diversity criterion is accomplished, the candidate vector *z *will join the *refset *only if

*f*(*z*) <*f*(*x*^*i*^)(1 - *ε*) ∀ *x*^*i *^∈ *R *    (30)

where *ε *is a small value defined by the user.

#### Refset regeneration

When all possible new combinations have been done and none of the generated vectors can replace any of the *elite *vectors, the algorithm can either stop or continue by regenerating the *refset*. The latter strategy is used in our algorithm. The worst *g elite *vectors (in terms of *fitness*) are deleted. New diverse vectors are generated (see above) and the *refset *is refilled according to a diversity criterion as the one described in the *refset *formation.

Normally *g *= *b*/2 but in aggressive implementations it can be set to *b *- 1 (i.e. all the vectors in the *refset *except the best one are deleted).

A new strategy for regenerating the *refset *has been implemented in SSm. Because the classical diversity criterion based on Euclidean distances described above does not ensure that the search will be performed along the different dimensions of the space. In our new strategy the vectors refilling the *refset *are chosen to maximize the number of relative directions defined by them and the existing vectors in the *refset*.

After deleting the *g *worst solutions the *refset *is (*b *- *g*) × *n *dimensional. Again, we compute *h *as the number of existing vectors in the current *refset *thus when the regeneration starts *h *= *b *- *g*.

A new matrix *M *containing the vectors that define the segments formed by the best vector in *refset *and the rest of vector in it is defined as

*M*^*h *-1 × *n *^= *x*^1 ^-*x*^*k *^∀ *k *= 2,3,...,*h *    (31)

with *x*^1 ^being the best element not deleted in *refset *in terms of *fitness *and *x*^*k *^the rest of the elements in it (note that the *refset *is ordered according to *fitness*).

For every diverse vector *x*^*v *^with *v *∈ [1, 2,...,*m*] to join the *refset *in the regeneration phase a vector *P *of scalar products is also defined:

*P*^*v *^= (*x*^1 ^- *x*^*v*^)·*M*^*T *^    (32)

where *x*^1 ^is again the best not deleted element in *refset *and *M*^*T *^is the transpose matrix of *M*. For every *x*^*v *^the maximum value of its vector *P*^*v *^is computed as *msp*(*x*^*v*^).

The solution *v *∈ *x*^*v *^will join the *refset *in the regeneration phase if

*msp*(*v*) = *min*{*msp*(*x*^*v*^)}     (33)

with *v *∈ *x*^*v*^. In this stage, the value of *h *is increased one unit and the process continues until *h *= *b*. The application of this strategy results in a maximum diversity in search directions on the regenerated *refset*.

#### Local search – filters

Local searches are carried out from different vectors as initial points to accelerate the convergence to the minima as shown in Figure 1. The user can use a different set of local solvers (see list above) to solve their problems. When a local (maybe global) solution provided by a local search outperforms the vector used as initial point to start the local search in terms of *fitness*, the former replaces the latter and becomes a member to join the *refset*. Otherwise, the solution obtained in the local search is discarded.

To avoid doing too many local searches or start from different initial points that might provide the same local solutions, two filters are implemented in the routine. The first one is a *merit *filter that takes into account the *fitness *of the vectors so that a local search is not started from bad vectors in terms of *fitness*. The other filter takes into account distances from initial points to the local solutions they provide, thus it avoids starting local searches from the area of influence of already found minima.

In principle, both filters must be passed to start a local search, but depending on the characteristics of the problem, any of them (or both) can be deactivated. Furthermore, they can be relaxed if no vectors passing them are found after a number of consecutive iterations.

#### Stopping criterion

The stopping criterion is taken as a combination of three conditions:

• maximum number of evaluations exceeded

• maximum computational time exceeded

• value to reach of the cost function satisfied

By default, the algorithm will stop when any of these conditions is satisfied.

## Authors' contributions

MRF performed the parameter estimation and the identifiability analysis and drafted the manuscript. JAE developed the novel metaheuristic and assisted in the preparation of the manuscript. JRB conceived of the study and participated in its design and coordination. All authors read and approved the final manuscript.
